# Safety assessment of a proprietary fenugreek mucilage composition (FenuMat®): Acute and subchronic toxicity studies

**DOI:** 10.1016/j.toxrep.2025.102187

**Published:** 2025-12-18

**Authors:** Megha Kelenchery Ganesh, Rani K. Cherian, Syam Das Sivadasan, Manu Aryan, Krishnakumar Illathu Madhavamenon

**Affiliations:** a CARe KERALAM Ltd, Thrissur, Kerala, India; b Akay Natural Ingredients Pvt Ltd, Cochin, Kerala, India; c Department of Immunology, Amala Cancer Research Centre Thrissur, Kerala, India

**Keywords:** FenuMat®, Fenugreek gum, Fenugreek mucilage, Galactomannan, Soluble dietary fiber, Toxicity

## Abstract

Fenugreek (*Trigonella foenum-graecum*) seeds and their extracts are popular culinary ingredients and nutraceuticals. The seed mucilage contains galactomannan, a soluble dietary fiber with prebiotic potential and divergent therapeutic effects. A proprietary fenugreek galactomannan preparation (F-GM), FenuMat®, functions as a natural self-emulsifying hydrogel, enhancing nutrient delivery and bioavailability. Although fenugreek seed safety is well documented, the safety evaluation of FenuMat® is warranted to ensure its compliance with regulatory standards. Hence, the present study evaluated the safety of FenuMat® in adult Wistar rats of both sexes, following OECD guidelines. In the acute oral toxicity study (14 days; OECD Guideline 423), no treatment related adversities were observed, indicating an LD₅₀ above 2000 mg/kg body weight. The subchronic toxicity study (OECD Guideline 408, repeated dose; 90 days) at doses of 250, 500 and 1000 mg/kg b.wt. revealed no significant alterations in hematological or biochemical parameters, or in food and water intake. However, significant reductions in serum glucose levels (at 500 mg/kg and 1000 mg/kg b.wt.) and LDL cholesterol levels (at 1000 mg/kg b.wt.) were observed. Histopathological evaluation revealed no morphological abnormalities in the major organs. Terminal autopsy revealed consistent relative organ weights and no treatment-related histopathological alterations. The high-dose recovery group (1000 mg/kg b.wt.) exhibited no mortality or adverse effects, with hematological and biochemical parameters comparable to controls, indicating a no-observed-adverse-effect level (NOAEL) of 1000 mg/kg b.wt. Further, the Ames test on four *Salmonella triphimurium* strains, with and without metabolic activation, demonstrated no mutagenic potential, indicating FenuMat®’s suitability for human use.

## Highlights

• Fenugreek seed mucilage (galactomannan) as a ‘self-emulsifying natural hydrogel’ (FenuMat®).
• Fenugreek galactomannan (F-GM) is a debitterized soluble dietary fiber.
• Acute toxicity study indicated the safety of F-GM with an LD_50_ as > 2000 mg/kg body weight.
• Sub-chronic toxicity study did not show any toxicity and indicated NOAEL as 1000 mg/kg body weight.
• Genotoxicity studies indicated the non-mutagenicity of F-GM.

## Graphical Abstract

**Figure:**
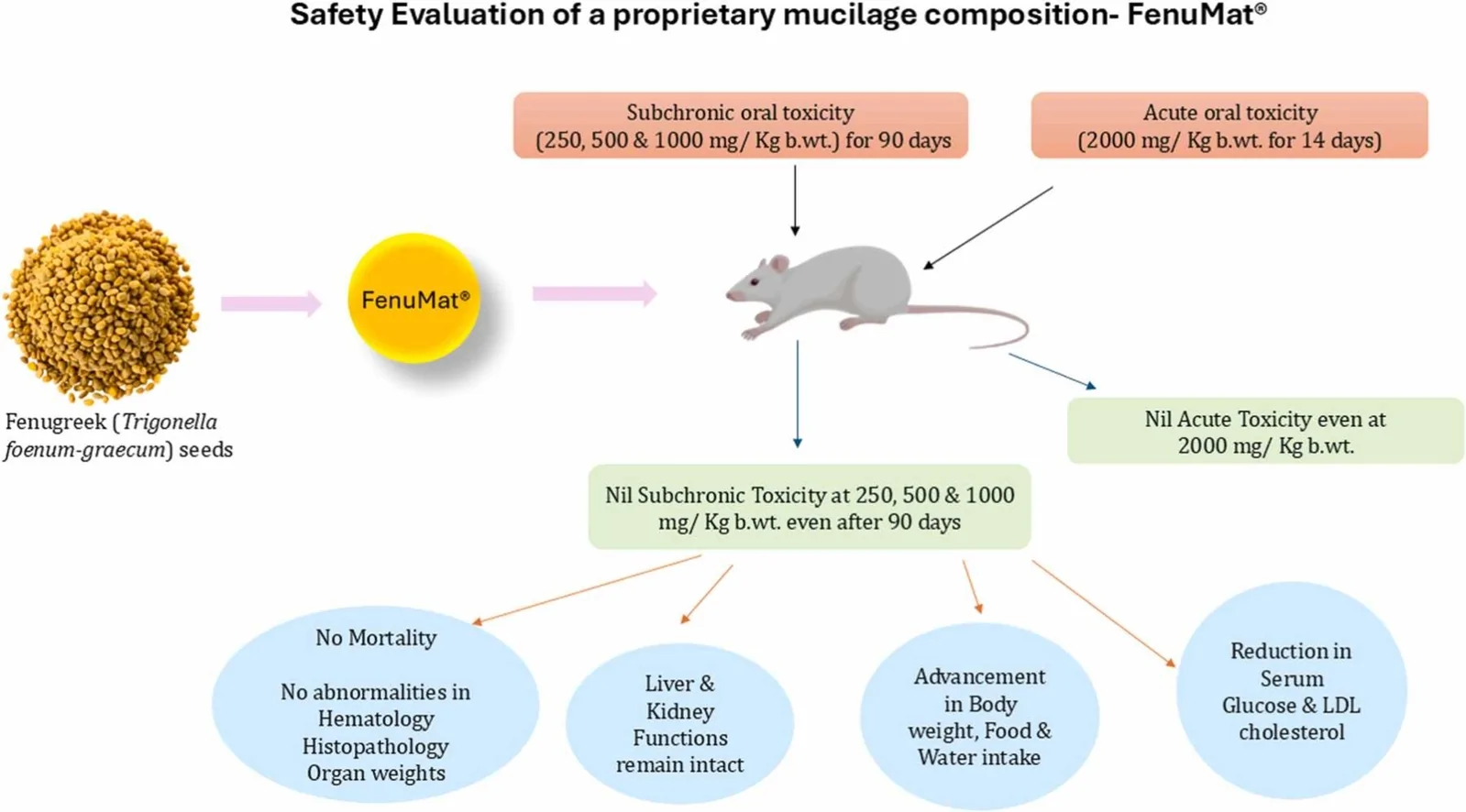

## 1 Introduction

Over the past three decades, scientific research has highlighted the health benefits of standardized botanical extracts in managing metabolic, neurodegenerative, cardiovascular and age-related diseases, as well as cancer [1], [2]. Fenugreek (*Trigonella foenum-graecum Linn.*), from the *Fabaceae* family, is widely used both as a spice and medicinal herb, with effective potential in regulating hormonal balance, metabolism, and aging [3], [4]. International food safety authorities, such as the United States Food and Drug Administration (USFDA) and the European Food Safety Authority (EFSA), have included fenugreek seeds as GRAS (Generally Recognized as Safe) and approved them globally as a food flavor additive. The global market for fenugreek is growing rapidly, driven largely by nutraceutical demand, with India as the lead producer with 250,000 metric tons annually [5], [6]. Fenugreek seeds are rich source of proteins (23–26 %), fats (6–7 %), and dietary fiber (45–50 %) along with bioactive phytochemicals, including saponins, alkaloids, flavonoids, amino acids, and micronutrients (vitamins and minerals) [7]. About half of the seed’s dry weight is dietary fiber, evenly categorized as insoluble fibers like cellulose and lignans, and soluble fibers primarily composed of galactomannans, known as fenugreek gum or mucilage [7], [8].

Galactomannans from various plants, including fenugreek, has demonstrated various therapeutic benefits, such as modulation of glucose metabolism, lipid-lowering effects, and gastrointestinal protective properties. Fenugreek galactomannans (F-GM) are composed of a *β*-(1 →4)-D-mannan backbone substituted with D-galactose units linked via *α*-(1 →6) glycosidic linkage (Fig. 1), with approximately equal numbers of mannose and galactose residues (180 and 190 units, respectively) and a molecular weight of about 30 kDa. This unique branched structure contributes to its high viscosity and gel-forming ability, which is central to its mucoadhesive properties. The galactose side chains interact with mucin glycoproteins in the gastrointestinal tract, promoting adhesion to the mucosal surface. This mucoadhesion can enhance the retention time of F-GM at the site of action, facilitating sustained interaction with intestinal enzymes and epithelial cells. As a soluble dietary fiber, F-GM has shown promising roles on glucose regulation and insulin sensitivity. Clinical studies report significant reductions in fasting blood glucose levels and glycated hemoglobin (HbA1c) in type 2 diabetes patients and a recent meta-analysis identifies galactomannans as the most effective soluble dietary fiber for managing type 2 diabetes [9], [10], [11].

**Fig. 1:**
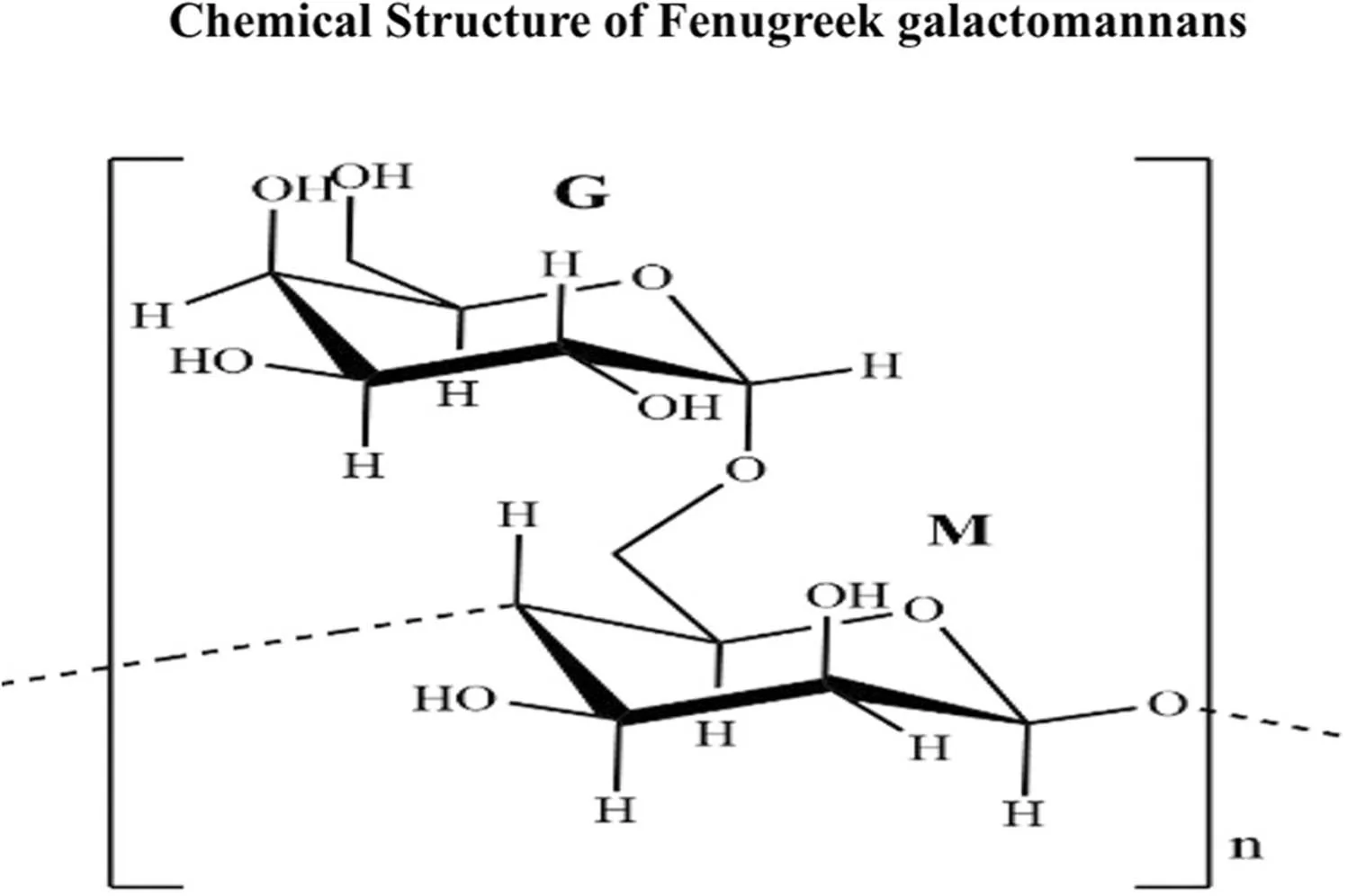
Chemical Structure of Fenugreek galactomannans [8].

F-GM modulates NF-κB and Keap1/Nrf2 pathways, providing protection against diabetic nephropathy and liver damage [12], [13]. It also reduces absorption of cholesterol and bile salts, leading to reductions in total cholesterol, LDL cholesterol, and triglyceride levels [14], [15], [16]. Additionally, F-GM reduces appetite, boosts calorie expenditure [17], [18], and may help manage heartburn and gastroesophageal reflux disease (GERD) [19], [20]. As a stable, non-digestible prebiotic fiber, F-GM favors gut microbiota balance and promotes short- chain fatty acid production [21], [22], [23].

Among the various innovations, the notable one is FenuMat®, a patented, de-bitterised fenugreek mucilage composition developed to enhance the solubility, stability, and bioavailability of hydrophobic phytonutrients including curcumin [25], [26]. FenuMat® functions as a natural, self-emulsifying hydrogel scaffold and has been utilized in hybrid-hydrogel systems designed for targeted delivery of herbal extracts, micronutrients, and vitamins [27], [28], [29]. Besides drug delivery, fenugreek mucilage can be applied in nanotechnology, including nanoparticle stabilization and edible food packaging films, as well as in tissue engineering and cosmetic formulations, highlighting its versatility as a natural hydrocolloid [7], [8], [24], [30].

Besides these promising applications and the already established safety of fenugreek seeds, specific safety evaluations of FenuMat® are essential to ensure its compliance with regulatory standards for human consumption. Previous research on low molecular weight fenugreek galactomannans have showcased an LD_50_ greater than 2000 mg/kg body weight and no-observed-adverse-effects level (NOAEL) at 1000 mg/kg body weight in repeated-dose toxicity studies, along with the absence of genotoxicity and developmental toxicity concerns [23], [31], [32]. However, considering FenuMat®’s unique processing and functional properties, detailed toxicological evaluations are necessary to confirm its safety profile.

Hence, the present study aims to evaluate the acute (14 day) and subchronic (90-day repeated-dose) oral toxicity, as well as the genotoxicity, of FenuMat® in Wistar rats. The evaluations were conducted as per internationally accepted guidelines of the Organization for Economic Co-operation and Development (OECD) to provide comprehensive and scientific safety data supporting the utilization of FenuMat® as a functional food ingredient and nutraceutical for human intervention.

## 2 Materials and methods

### 2.1 Test substance

The proprietary material of FenuMat®-mucilage (FGS-002P) was obtained from Akay Natural Ingredients, Cochin, Kerala, India, along with a certificate of analysis and material safety data sheet. The test item, FGS-002P, was an off-white, fine powder with an approximate sample quantity of 25 g and was stored at room temperature throughout the study. Being a universally accepted and routinely used vehicle in oral (gavage) toxicity studies along with the capability of test substance to form a good suspension, distilled water was used as the vehicle for formulation preparation in the present study.

### 2.2 Animals

Adult Wistar rats of both sexes (200–250 g) were used for the toxicological studies. The animals were procured from Small Animal Research Centre, Department of Toxicology & Pharmacology, CARe Keralam Ltd, Kerala, India, and were acclimatized for a period of 14 days in ventilated cages. They were housed in the animal house facility in an air-conditioned room maintained at 22 ± 3^0^C with a relative humidity of 60–70 % and a 12 h light and dark cycle. All animal experiments were carried out in strict accordance with the ethical norms approved by the Institutional Animal Ethics Committee (IAEC), recognized by the Committee for the Purpose of Control and Supervision of Experiments on Animals (CPCSEA), Government of India (Registration No: CKL/TOX/IAEC/2023–2/202). The animals were provided with a pellet diet and water *ad libitum*.

### 2.3 Toxicity studies

#### 2.3.1 Acute toxicity (14 days) study

Acute toxicity studies were initially performed in accordance with OECD Guideline 423 to verify whether FGS-002P produced any toxic effects when supplemented at the maximum recommended concentration [33]. FGS-002P was suspended in water and orally administered as a single dose at the limit dose of 2000 mg/kg body weight to five nulliparous female Wistar rats. All the animals were observed for mortality and for clinical and behavioral signs at 10, 30, 60, 120, 240, and 360 min post-dose, and thereafter twice daily for mortality and once daily for clinical signs during the study period of 14 days. On day 15, all animals were euthanized by CO_2_ inhalation and were subjected to gross necropsy. All gross pathological changes were recorded for each animal.

#### 2.3.2 Sub chronic oral toxicity study

A Sub-chronic toxicity study was conducted in accordance with OECD Guideline No. 408 [34]. Sixty Wistar rats (30 males and 30 females) with an average body weight of 200–250 g were divided into six groups, each consisting of five males and five females, as follows;

Group I – Control – 1 mL distilled water/100 g body weight

Group II – Control Recovery (Control R) – 1 mL distilled water/100 g body weight

Group III – Low dose (LD) – FGS-002P at 250 mg/kg body weight

Group IV – Medium dose (MD) – FGS-002P at 500 mg/kg body weight

Group V – High Dose (HD) – FGS-002P at 1000 mg/kg body weight

Group VI – High Dose Recovery (HD-R) – FGS-002P at 1000 mg/kg body weight

FGS-002P was suspended in distilled water and orally administered to the animals using an oral feeding needle in such a way that all the animals received same volume of vehicle. All treated rats were observed for mortality, clinical signs and adverse reactions during the 90-days study period, whereas recovery groups (Control R & HD-R) were observed for 119 days (including the reversal period of 28 days). The body weight, food and water consumption were determined every week up to 90 days by measuring the left-over feed after 24 h and was noted for a single cage of five animals. At the end of the study period, the animals were euthanized by CO_2_ inhalation followed by cervical dislocation to ensure death, based on ethical guidelines. Organs were collected, washed with saline, weighed, and examined for structural abnormalities.

Blood samples from all experimental animals was collected in EDTA coated vials and analyzed for red blood cells (RBCs) count, white blood cells (WBCs) count, platelet count and hemoglobin (Hb) content using a hematological analyzer (Model-Diatron, Wein, Austria). For biochemical analysis, serum was separated by centrifugation at 3000 rpm for 10 min at −4℃ and was stored at −20℃. Total bilirubin was determined as per the Pearlman method [35]. Alkaline phosphatase (ALP), serum glutamate oxaloacetate aminotransferase (SGOT) and serum glutamate pyruvate aminotransferase (SGPT) were estimated using kinetic method kits from M/s Raichem, India, using a Micro lab 300 auto-analyzer (Merck, Mumbai, India). Albumin was determined by its reaction with bromocresol green and the total protein concentration was determined by the Biuret method [36]. Kidney function markers, such as creatinine and blood urea, were estimated by Jaffe’s kinetic and urease methods respectively [37]. The total cholesterol was estimated as per the method of Deeg et al., [38], triglycerides by the GPO–PAP (Glycerol Phosphate Oxidase-Peroxidase 4-Aminoantipyrine method) method [39], HDL cholesterol by precipitation with phosphotungstic acid and LDL cholesterol by the equation; LDL = total cholesterol − (HDL + VLDL) [40]. Serum uric acid was determined using a commercial kit based on the manufacturer’s protocol (Thermo Fisher Scientific, USA).

#### 2.3.3 Histopathological studies

Necropsy was performed in the presence of a veterinary doctor, and all animals were visually examined for any abnormalities. All the organs were separated, and their individual weights were recorded. The weights of the brain, liver, heart, kidney, lungs, ovary, testis and spleen were measured and expressed relative to the final body weight. Tissue samples were fixed in 10 % formalin, and paraffin embedded tissues were cut into slices of 2–4 μm and stained with hematoxylin and eosin for histopathological examinations with an optical microscope at 100**×** magnification (Olympus-Magnus trinocular microscope, Tokyo, Japan).

#### 2.3.4 Mutagenicity study

The mutagenic potential of F-GM was assessed using the AMES test (Bacterial Reverse Mutation test) following the OECD Guideline No. 471. The assay employed four histidine-dependent *Salmonella typhimurium* strains: TA98, TA100, TA1535 and TA102. Each strain was tested in triplicate at five doses of F-GM (0.128, 0.320, 0.800, 2, 5 mg/plate) using plate incorporation method, both in the presence and absence of an exogenous metabolic activation system. Two types of controls were use; (1) Positive control (2.5 μg sodium azide/plate dissolved in sterile distilled water), (2) Negative control (Vehicle control; sterile distilled water). The *in vitro* metabolic activation of the mutagen was facilitated using the metabolic activation system referred to as S9 mix. This system comprises of rat liver microsomal enzyme cytochrome P450 with suitable cofactors and 2-acetamidofluorene (20 μg) as the positive control. To perform the test, agar plates were prepared by pouring a mixture encompassing the specific bacterial strain, the test substance (F-GM), the S9 mix, and soft agar supplemented with trace amounts of histidine. Following incubation for 48 h at 37 °C, the revertant bacterial colonies were counted using a colony counter. The test substance was considered mutagenic if it induced a three-fold increase in the revertant colonies compared to the negative control. Reduction in revertant count/ thinning or absence of the background lawn indicated cytotoxicity of the test substance.

### 2.4 Statistical analysis

Data on body weight, food intake, organ weights, hematological, and clinical chemistry parameters were analyzed using SPSS software (version 25.0; IBM Corp., NY, USA). Results are expressed as mean ± standard error of the mean (SEM). Statistical comparisons between control and treatment groups were performed by one-way analysis of variance (ANOVA), followed by appropriate post hoc test (Dunnett’s multiple comparison test) to determine intergroup differences. The assumptions of normality and homogeneity of variance were evaluated prior to ANOVA. Data from FenuMat® treated animals were compared with that of the control group, and differences were considered statistically significant at p < 0.05.

## 3 Results

### 3.1 Acute toxicity studies of FGS-002P (14 days)

Oral administration of FGS-002P at a dose of 2000 mg/kg body weight did not result in any mortality or adverse effects during the 14-day study period, and no abnormal clinical signs were observed in Wistar rats. Body weight and food consumption remain unchanged, in comparison with the untreated control group. Necropsy at the end of study did not reveal any gross pathological abnormalities, suggesting that FGS-002P at the tested dose of 2000 mg/kg/day, is safe for Wistar rats (Supplementary tables, S1-S4).

### 3.2 Subchronic toxicity studies FGS-002P (90 days)

Animals categorized as treated and control groups, of both sexes, were examined daily for 90 days, to evaluate general appearance, behaviour, signs of toxicity, morbidity, and mortality. No mortality was observed during the study period. All animals in both the treated and control groups remained active and healthy throughout the study period.

#### 3.2.1 Effect on body weight

The weight gain among both male and female animals in the untreated and treated groups was normal. During the 90-day study period, the body weight (g) of untreated control male animals increased from 211 ± 3.68–325 ± 10.39 g, with an average growth rate of 1.16 ± 0.03 g/day, while that of females was 0.87 ± 0.02 g/day (increased from 201 ± 2.45–250 ± 21.27 g). A similar trend in body weight was observed among the FGS-002P treated (250, 500 and 1000 mg per kg body weight) animals as well. The observed weight gains for the highest tested dose of 1000 mg/kg body weight for male rats were 1.11 ± 0.08 per day and that of female rats increased from 224 ± 3.56–293.5 ± 12.94 (0.91 ± 0.01 g per day). Following a 28-day observation period for the high dose recovery group, animals of both sexes showed no abnormal clinical signs (general behaviour) or mortality compared with the control group. Furthermore, no significant differences in body weight were observed between the male and female recovery groups and their respective control groups (Fig. 2 A and 2B).

**Fig. 2:**
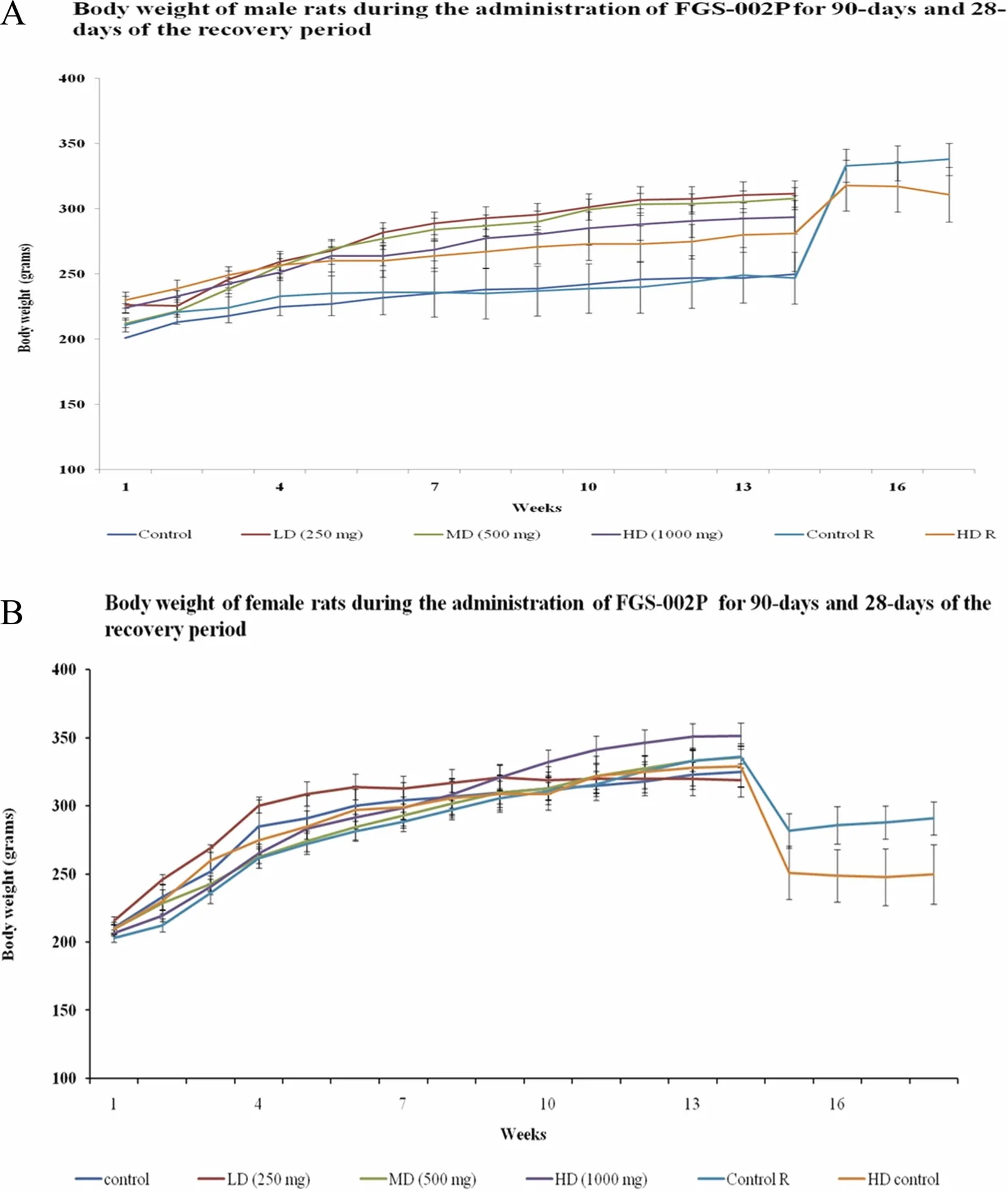
Body weight of rats during the administration of FGS-002P.

#### 3.2.2 Effect on food and water consumption

Administration of FGS-002P at doses of 250 mg/kg body weight (Group III), 500 mg/kg body weight (Group IV) and 1000 mg/kg body weight (Group V) did not produce any significant difference in food consumption among male and female rats compared with the untreated group. The average food intake of male rats was nearly 2.62 g/animal/day, while that of females was 2.21 g/animal/day (Fig. 3 A and 3B). Water consumption of the FGS-002P treated animals also remained unchanged compared with the untreated controls. Following a 28-day observation period for the high dose recovery group, animals of both sexes showed no substantial differences in food and water consumption relative to the control recovery group (Fig. 4 A and 4B).

**Fig. 3:**
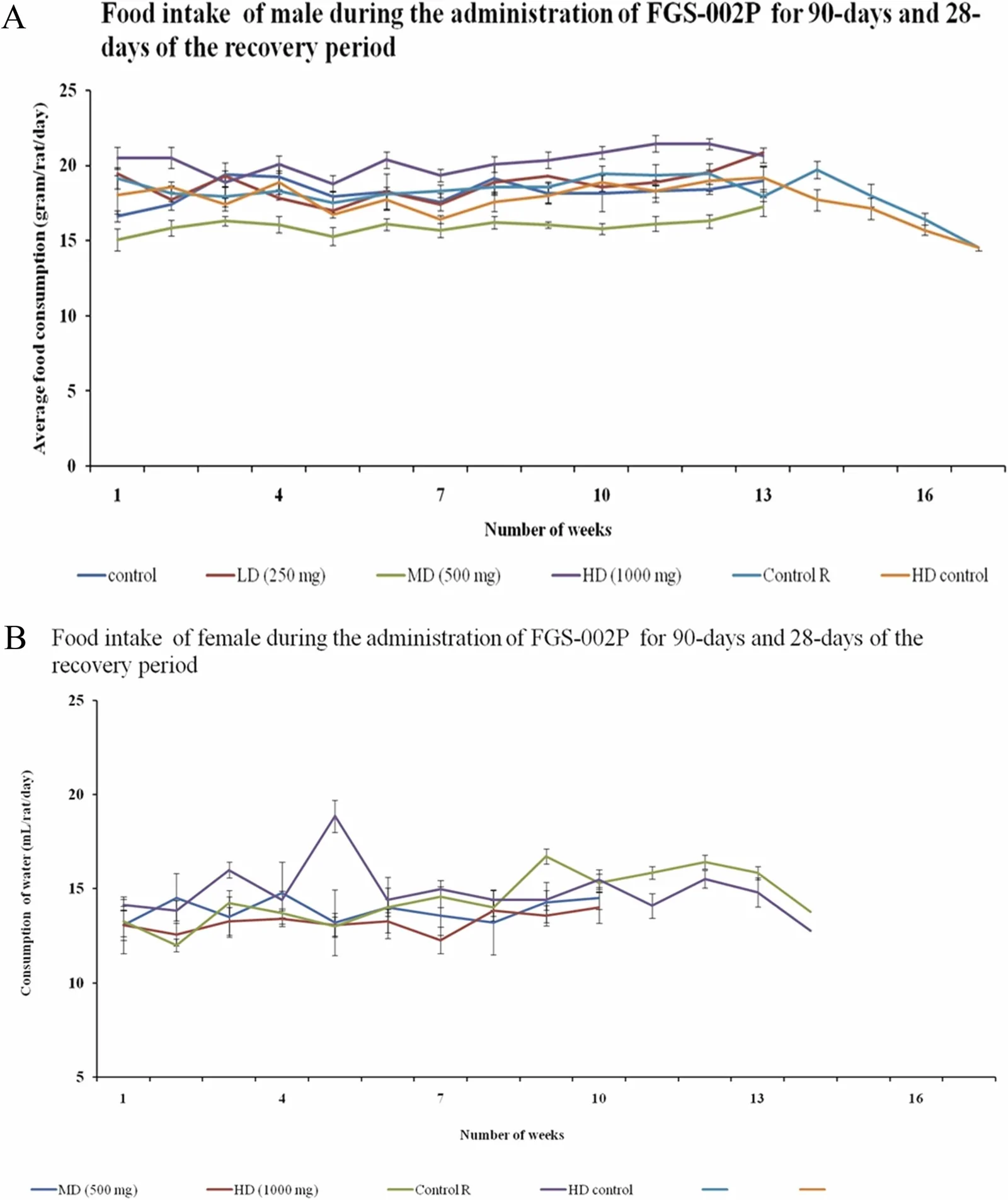
Food intake of rats during the administration of FGS-002P.

**Fig. 4:**
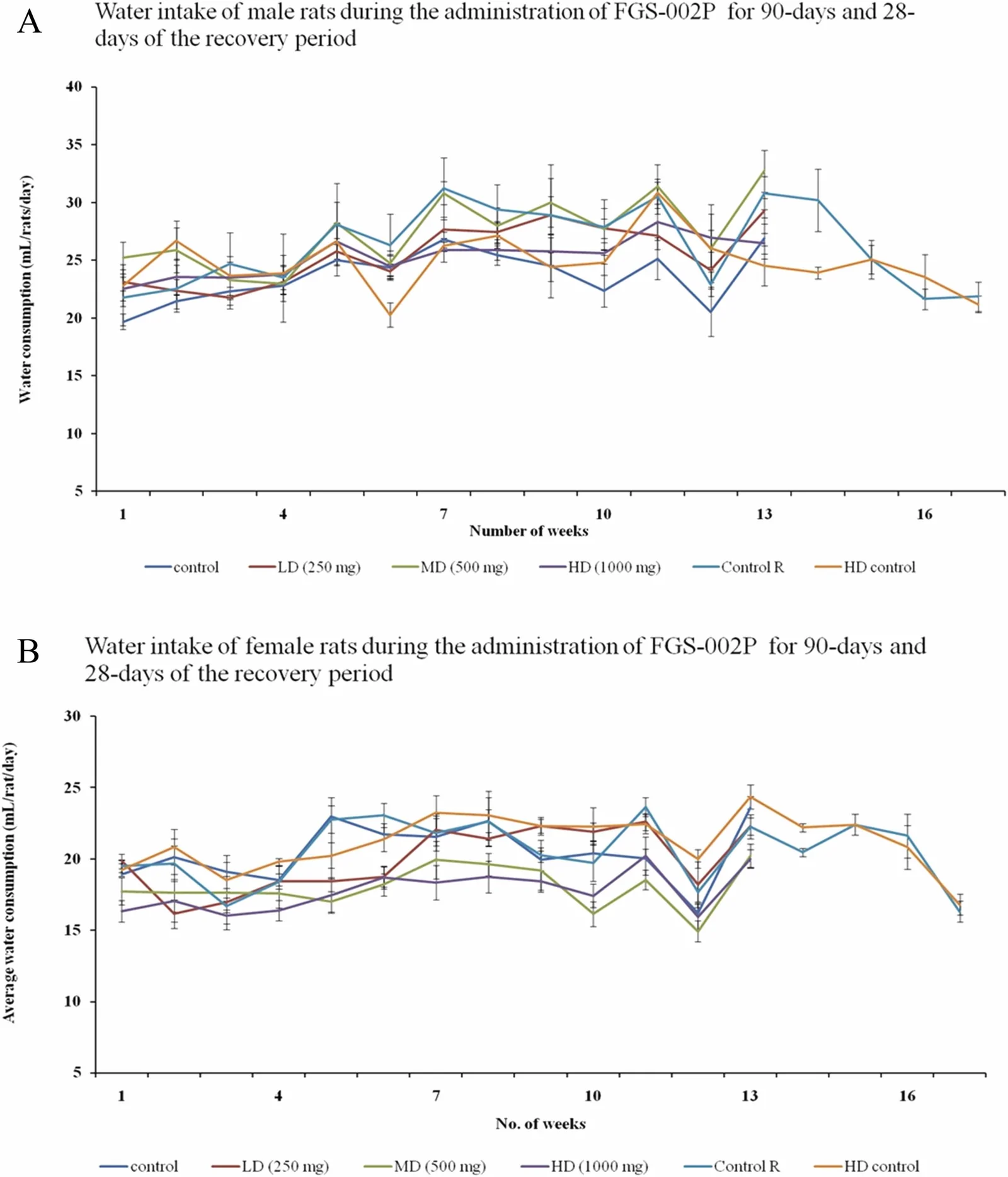
Water intake of rats during the administration of FGS-002P.

#### 3.2.3 Necropsy and organ weights

The relative organ weights (liver, kidney, heart, spleen, lungs, brain, ovary and testis) of FGS-002P treated animals showed no significant changes compared with the untreated control group of male and female animals. Necropsy of the treated animals showed a normal appearance of the organs and tissues (Table 1).

**Table 1:** Effect of 90 days of repeated dose of fenugreek on relative organ weight.

**Groups**	**Liver**	**Kidney**	**Spleen**	**Heart**	**Lungs**	**Brain**	**Ovary**
**Male**							
Control	3.10 ± 0.18	0.60 ± 0.03	0.14 ± 0.01	0.30 ± 0.02	0.53 ± 0.04	0.39 ± 0.01	0.85 ± 0.05
Control R	2.80 ± 0.21	0.78 ± 0.08	0.14 ± 0.02	0.39 ± 0.04	0.56 ± 0.0	0.42 ± 0.02	0.85 ± 0.09
LD (250 mg)	3.07 ± 0.18	0.63 ± 0.02	0.15 ± 0.01	0.31 ± 0.01	0.58 ± 0.01	0.29 ± 0.01	0.77 ± 0.03
MD (500 mg)	3.17 ± 0.16	0.67 ± 0.03	0.15 ± 0.01	0.31 ± 0.01	0.58 ± 0.01	0.29 ± 0.01	0.80 ± 0.04
HD (100 mg)	3.21 ± 0.11	0.69 ± 0.02	0.15 ± 0.007	0.33 ± 0.01	0.59 ± 0.02	0.59 ± 0.02	0.89 ± 0.03
HD R	4.58 ± 0.34	1.12 ± 0.03	0.26 ± 0.03	0.56 ± 0.02	0.88 ± 0.04	0.68 ± 0.05	1.35 ± 0.06
**Female**							
Control	3.71 ± 0.27	0.75 ± 0.05	0.19 ± 0.02	0.43 ± 0.04	2 ± 0.10	1.37 ± 0.03	0.10 ± 0.03
Control R	3.71 ± 0.33	0.78 ± 0.08	0.19 ± 0.01	0.44 ± 0.04	0.76 ± 0.03	0.50 ± 0.04	0.30 ± 0.11
LD (250 mg)	3.00 ± 0.13	0.59 ± 0.04	0.14 ± 0.010	0.30 ± 0.01	0.58 ± 0.03	0.38 ± 0.009	0.16 ± 0.07
MD (100 mg)	2.53 ± 0.17	0.59 ± 0.04	0.13 ± 0.006	0.29 ± 0.01	0.59 ± 0.02	0.42 ± 0.02	0.18 ± 0.16
HD (1000 mg)	8.57 ± 0.46	0.60 ± 0.05	0.16 ± 0.008	0.33 ± 0.02	0.67 ± 0.04	0.49 ± 0.02	0.06 ± 0.01
HD R	3.25 ± 0.19	0.70 ± 0.10	0.70 ± 0.10	0.35 ± 0.01	0.58 ± 0.04	0.48 ± 0.01	0.26 ± 0.16

Values are mean ± standard error mean, expressed as the percentage of organ weight/100 g of body weight. Five animals/sex/group, unless otherwise specified

#### 3.2.4 Hematological parameters

As shown in Table 2, FGS-002P treated groups of both sexes showed no significant changes in hematological parameters. Parameters such as hemoglobin, WBC, RBC, and platelet counts in treated groups were in the normal range when compared to the control group. In the same way, high dose recovery group animals also showed no significant alterations in hematology parameters (Table 2).

**Table 2:** Effect of fenugreek administration on hematological parameters of Wistar rats.

Measured parameters	**Control**	Control R	LD (250 mg)	MD (500 mg)	HD (1000 mg)	HD R (1000 mg)
**Male**						
RBC count (10^6^ cell/µL)	8.21 + 0.31	7.84 ± 0.31	7.96 ± 0.17	7.77 ± 0.19	7.69 ± 0.15	7.84 ± 0.19
WBC count (10^3^ cell/µL	7.36 + 0.40	7.92 ± 0.61	7.41 ± 0.57	7.68 ± 0.34	6.69 ± 0.12	8.94 ± 1.03
Lymphocytes (10^3^ cell/µL)	5.84 ± 0.28	6.38 ± 0.52	5.88 ± 0.47	5.99 ± 0.27	5.22 ± 0.15	7.34 ± 0.80
Granulocytes (10^3^ cell/µL)	0.82 ± 0.07	0.76 ± 0.08	0.77 ± 0.05	0.82 ± 0.05	0.81 ± 0.04	0.82 ± 0.08
MID cells (10^3^ cell/µL)	0.7 ± 0.14	0.78 ± 0.13	0.76 ± 0.08	0.87 ± 0.07	0.66 ± 0.04	0.78 ± 0.17
Hb (g/dL)	14 ± 0.33	13.72 ± 0.26	14.89 ± 0.20	14.9 ± 0.32	13.88 ± 0.50	14.46 ± 0.43
Platelet count (10^3^ cell/µL)	829.2 ± 56.14	835.2 ± 44.5	772.5 ± 37.68	876.2 ± 21.48	831.8 ± 18.54	836.4 ± 25.2
**Female**						
RBC count (10^6^ cell/µL)	8.04 ± 0.31	7.78 ± 0.37	7.66 ± 0.21	8.00 ± 0.33	8.03 ± 0.26	7.89 ± 0.34
WBC count (10^3^ cell/µL)	7.5 ± 0.18	7.07 ± 0.39	7.01 ± 0.28	6.82 ± 0.18	7.08 ± 0.18	6.68 ± 0.28
Lymphocytes (10^3^ cell/µL)	5.92 ± 0.20	5.66 ± 0.36	5.5 ± 0.26	5.34 ± 0.17	5.57 ± 0.09	5.18 ± 0.34
Granulocytes (10^3^ cell/µL)	0.96 ± 0.08	0.82 ± 0.07	0.78 ± 0.05	0.84 ± 0.08	0.86 ± 0.04	0.68 ± 0.04
MID cells (10^3^ cell/µL)	0.62 ± 0.11	0.56 ± 0.06	0.73 ± 0.06	0.64 ± 0.05	0.65 ± 0.06	0.82 ± 0.12
Hb (g/dL)	14.04 ± 0.51	13.88 ± 0.47	13.96 ± 0.47	14.41 ± 0.37	13.78 ± 0.25	13.98 ± 0.24
Platelet count (10^3^ cell/µL)	805.8 ± 24.61	822 ± 35.38	829.9 ± 29.08	801.5 ± 20.03	772 ± 20.15	787.4 ± 39.10

The values are expressed as mean ± standard deviation. Five animals/sex/group unless otherwise specified.

#### 3.2.5 Serum biochemical parameters

Biochemical parameters in FGS-002P administered animals did not elicit any significant changes in comparison with the control group. Hepatic function markers such as SGOT, SGPT, ALP, total bilirubin, direct bilirubin, total protein, albumin and CRP remained unaltered in both sexes (Tables 3 A and 3B). Lipid profile also displayed no significant variation in total cholesterol, triglycerides, and HDL cholesterol levels among both male and female rats and were comparable to that of untreated control group. However, a decreasing trend in glucose levels was observed in animals treated with 500 mg/kg (p < 0.05) and 1000 mg/kg body weight of FGS-002P (p < 0.001). Furthermore, a significant decline (p < 0.05) in LDL cholesterol level was observed in male rats at the highest dose. Both sexes of the high dose recovery group animals also showed no significant change in biochemical parameters, as compared to the untreated control group. Renal function parameters such as urea, creatinine and uric acid, were comparable between treated and control animals in both sexes (Tables 4 A and 4B) (Table 3A) (Table 3B) (Table 4A) (Table 4B).

**Table 3A:** Effect of 90 days of repeated dose of fenugreek on Liver function markers and CRP on male Wistar rats.

Measured parameters	Dose group					
	**Control**	**Control R**	**LD** **250 mg**	**MD** **500 mg**	**HD** **1000 mg**	**HD R** **1000 mg**
ALT (U/L)	154.19 ± 5.94	153.98 ± 3.40	146.73 ± 5.51	151.30 ± 4.77	150.69 ± 2.58	150.55 ± 4.96
AST (mg/dL)	52.05 ± 0.97	57.69 ± 3.86	59.79 ± 2.81	55.13 ± 2.47	54.83 ± 2.18	58.24 ± 5.51
ALP (mg/dL)	143.46 ± 9.11	162.02 ± 5.46	153.35 ± 5.12	155.16 ± 4.06	153.45 ± 3.39	148.83 ± 6.31
T. Bilirubin (mg/dL)	0.44 ± 0.55	0.42 ± 0.03	0.41 ± 0.03	0.44 ± 0.03	0.43 ± 0.02	0.42 ± 0.03
D. Bilirubin (mg/dL)	0.175 ± 0.04	0.18 ± 0.02	0.15 ± 0.02	0.16 ± 0.02	0.19 ± 0.02	0.18 ± 0.03
Albumin (g/dL)	3.62 ± 0.13	3.77 ± 0.13	3.46 ± 0.09	3.42 ± 0.03	3.68 ± 0.22	3.77 ± 0.13
Total Protein (g/dL)	7.17 ± 0.23	6.84 ± 0.30	7.14 ± 0.12	6.91 ± 0.13	7.13 ± 0.11	6.96 ± 0.30
CRP (mg/dL)	1.24 ± 0.10	1.17 ± 0.05	1.29 ± 0.05	1.33 ± 0.05	1.33 ± 0.06	1.25 ± 0.12

The values are expressed as mean ± standard error mean; Five animals/sex/group, unless otherwise specified.

**Table 3B:** Effect of 90 days of repeated dose of fenugreek on Liver function markers and CRP on female Wistar rats.

Measured parameters	Dose group					
	**Control**	**Control R**	**LD** **250 mg**	**MD** **500 mg**	**HD** **1000 mg**	**HD R** **1000 mg**
ALT (U/L)	140.49 ± 5.56	147.85 ± 7.08	150.64 ± 4.89	143.58 ± 2.16	150.40 ± 2.72	147.86 ± 10.37
AST (mg/dL)	54.14 ± 2.48	52.55 ± 3.20	48.75 ± 2.41	52.00 ± 1.95	50.90 ± 2.05	55.42 ± 3.59
ALP (mg/dL)	146.62 ± 8.77	155.90 ± 3.94	151.85 ± 3.96	150.45 ± 3.08	155.87 ± 2.77	153.23 ± 5.88
T Bilirubin (mg/dL)	0.38 ± 0.03	0.34 ± 0.05	0.44 ± 0.03	0.37 ± 0.03	0.4 ± 0.03	0.36 ± 0.05
D Bilirubin (mg/dL)	0.14 ± 0.02	0.18 ± 0.04	0.11 ± 0.01	0.14 ± 0.02	0.15 ± 0.02	0.16 ± 0.02
Albumin (g/dL)	3.60 ± 0.27	4.23 ± 0.23	3.87 ± 0.13	3.65 ± 0.17	3.56 ± 0.03	3.94 ± 0.21
Total Protein (g/dL)	7.17 ± 0.23	7.31 ± 0.27	7.14 ± 0.12	6.91 ± 0.13	7.13 ± 0.11	7.24 ± 0.26
CRP (mg/dL)	1.26 ± 0.08	1.26 ± 0.05	1.35 ± 0.04	1.40 ± 0.05	1.37 ± 0.05	1.29 ± 0.06

The values are expressed as mean ± standard error mean; 5 animals/sex/group, unless otherwise specified.

**Table 4A:** Effect of 90 days of repeated dose of fenugreek on Lipid profile and renal function on male Wistar rats.

Measured parameters	Dose group					
	**Control**	**Control R**	**LD** **250 mg**	**MD** **500 mg**	**HD** **1000 mg**	**HD R** **1000 mg**
Glucose (mg/dl)	123.3 ± 2.40	103.78 ± 7.76	110.59 ± 2.99	108.4 ± 3.00*	106.58 ± 3.36*	94.44 ± 1.80
Cholesterol (mg/dl)	72.58 ± 2.46	69.72 ± 2.36	64.44 ± 1.96	69.63 ± 1.80	65.49 ± 2.46	70.84 ± 2.01
Triglycerides (mg/dl)	59.98 ± 3.38	65.46 ± 5.50	61.74 ± 3.19	66.08 ± 2.23	59.32 ± 2.02	56.76 ± 3.37
HDL (mg/dl)	34.96 ± 4.20	31.36 ± 2.66	36.13 ± 1.40	35.83 ± 1.36	38.53 ± 1.21	33.98 ± 1.15
LDL (mg/dl)	25.62 ± 5.79	25.26 ± 3.09	16.01 ± 1.40	20.58 ± 1.54	15.09 ± 2.66*	25.50 ± 0.82
Urea (mg/dL)	37.06 ± 1.66	34.28 ± 0.93	33.77 ± 1.21	40.04 ± 2.06	38.86 ± 1.85	38.32 ± 2.84
Creatinine (mg/dl)	0.44 ± 0.01	0.43 ± 0.02	0.44 ± 0.01	0.47 ± 0.01	0.45 ± 0.02	0.45 ± 0.06
Uric Acid (mg/dl)	3.61 ± 0.38	3.13 ± 0.24	2.99 ± 0.26	3.10 ± 0.24	3.22 ± 0.27	3.21 ± 0.27

The values are expressed as mean ± standard error mean; Five animals/sex/group, unless otherwise specified

**Table 4B:** Effect of 90 days of repeated dose of fenugreek on Lipid profile and renal function of female Wistar rats.

**Measured** **Parameters**	Dose group					
	**Control**	**Control R**	**LD 250 mg**	**MD 500 mg**	**HD 1000 mg**	**HD R 1000 mg**
Glucose (mg/dl)	117.98 ± 2.49	108.73 ± 5.11	109.53 ± 2.11	106.07 ± 2.13*	106.83 ± 3.25*	93.36 ± 5.73
Cholesterol (mg/dl)	72.08 ± 2.64	65.14 ± 3.43	64.44 ± 1.96	71.14 ± 2.15	67.06 ± 2.31	66.12 ± 2.90
Triglycerides (mg/dl)	59.72 ± 2.47	63.94 ± 5.14	61.47 ± 3.19	64.49 ± 3.46	61.97 ± 2.24	61.68 ± 3.82
HDL (mg/dl)	37.52 ± 2.07	34.58 ± 3.51	36.13 ± 1.40	38.03 ± 1.29	36.00 ± 1.51	34.44 ± 3.04
LDL (mg/dl)	22.61 ± 4.03	17.77 ± 2.47	16.01 ± 0.97	20.21 ± 1.29	18.66 ± 1.90	19.34 ± 2.24
Urea (mg/dl)	32.92 ± 2.95	30.92 ± 1.67	38.27 ± 1.35	32.56 ± 1.28	36.57 ± 1.49	34.18 ± 1.38
Creatinine (mg/dl)	0.41 ± 0.04	0.46 ± 0.03	0.55 ± 0.05	0.55 ± 0.05	0.46 ± 0.05	0.50 ± 0.09
Uric Acid (mg/dl)	2.80 ± 0.23	3.26 ± 0.27	3.13 ± 0.19	2.95 ± 0.25	3.43 ± 0.31	3.06 ± 0.40

The values are expressed as mean ± standard error mean; Five animals/sex/group, unless otherwise specified.

#### 3.2.6 Histopathological analysis

Comprehensive gross and histological examinations were conducted on multiple vital organs, comprising the liver, heart, lungs, brain, kidneys, spleen, gastrointestinal tract, ovaries, and testes, to evaluate the potential impacts of FGS-002P following a 90-day administration period. The analysis indicated the absence of adverse effects or any pathologically important variations in the tissue morphology or architecture of these organs. Findings in the treated groups were comparable to those observed in the control group throughout both the treatment phase and the subsequent recovery period. These results suggest that prolonged exposure to FGS-002P does not induce organ toxicity or histopathological damage in the examined tissues (Fig. 5 A and 5B).

**Fig. 5:**
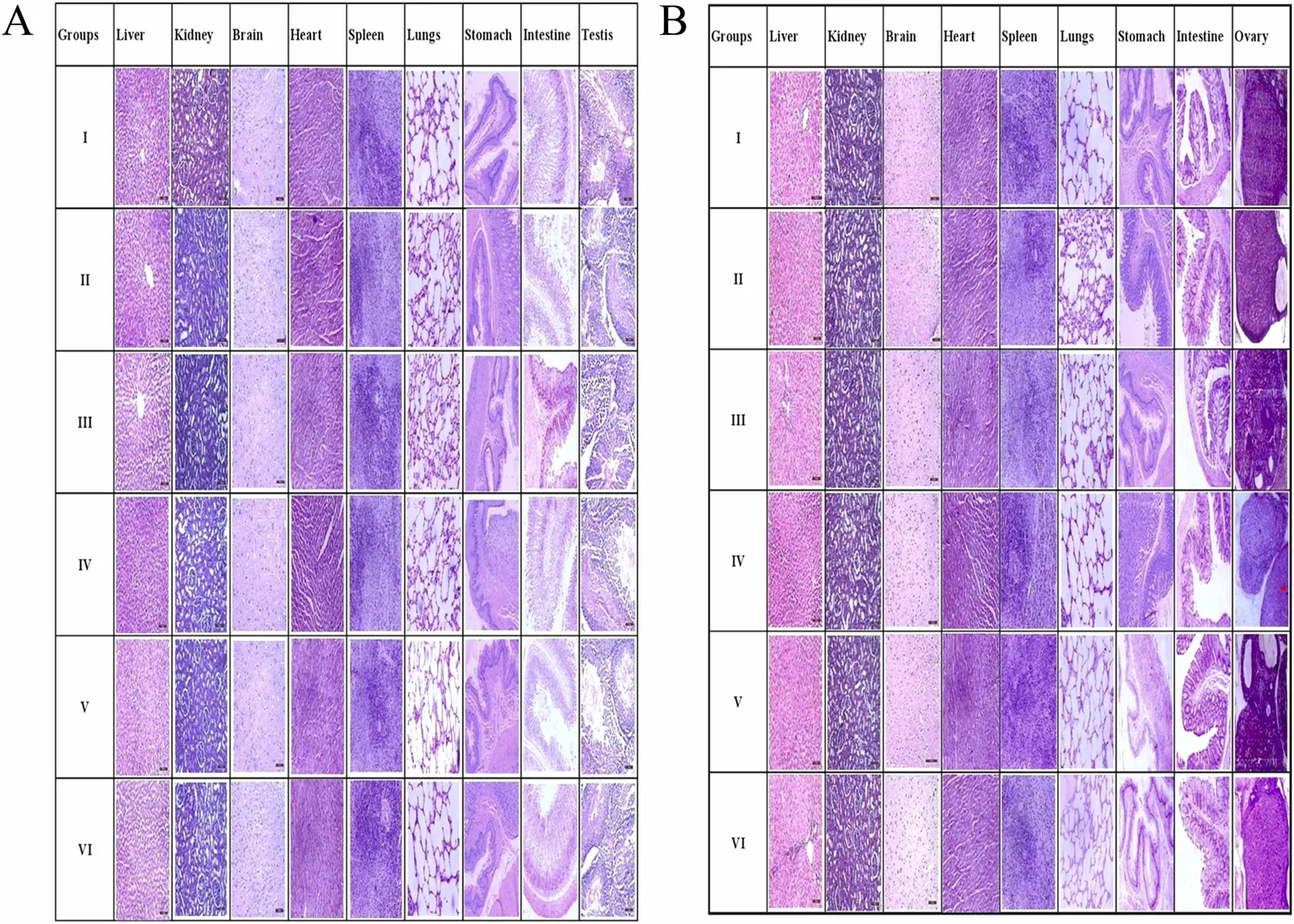
Histopathological images of rat organs.

### 3.3 Mutagenicity

F-GM did not produce any significant increase in the number of revertant colonies in any of the tested *Salmonella typhimarium* strains, either in the presence or absence of metabolic activation (S9 mix), at concentrations up to 5 mg/ plate when compared to the negative control. In contrast, positive controls comprising known mutagens induced a marked increase in the mean number of revertant colonies across all strains, while the vehicle control is devoid of such an enhancement (Table 5). Results from the present study indicates that F-GM does not elicit dose dependant mutagenicity, with or without metabolic activation.

**Table 5:** Results of mutagenicity as obtained from Ames Test.

Con. (mg/plate)	TA98	TA100	TA102	TA1535
	Mean ± SD	Mean ± SD	Mean ± SD	Mean ± SD
Negative Control (DW)	31 ± 4.36	130.33 ± 8.08	273.67 ± 24.58	22.33 ± 2.52
0.128	29.33 ± 2.89	126 ± 6.24	272 ± 38.97	24 ± 6.08
0.320	35.00 ± 7.00	132.00 ± 7.55	272 ± 43.51	23 ± 5.57
0.800	26.33 ± 8.74	139.33 ± 2.52	335.67 ± 119.87	25 ± 7.94
2.000	23.33 ± 4.16	125.33 ± 3.21	245.67 ± 24.44	24 ± 8.54
5.000	20.33 ± 9.07	126.00 ± 3.61	244.33 ± 14.57	17.33 ± 7.77
Positive Control (μg/ plate)	448.67 ± 44.77	866.33 ± 8.33	997 ± 122.39	439.33 ± 41.59

The values are expressed as mean ± standard error mean. Each strain was tested in triplicate.

## 4 Discussion

Dietary fibers are increasingly being utilized in food and nutrition (dietary supplements/ nutraceuticals) owing to their essentiality for optimized health and diverse pharmacological benefits. However, establishing safe and effective dosage is crucial. To ensure safety, regulatory guidelines to conduct safety assessments of bioactive substances under Good Laboratory Practice (GLP) have been established by the OECD. In the present research, we present the acute and subchronic toxicity evaluations of a proprietary fenugreek dietary fiber composition, FenuMat®-conducted based on the OECD guidelines. FenuMat® functions as an effective hydrogel matrix with notable health benefits, biocompatibility, and prebiotic potential. In drug delivery systems, hydrogels have been demonstrated to protect drug molecules from the gastrointestinal environment while facilitating controlled release at target sites [41].

The significance of FenuMat-fiber mucilage in food and nutrition is supported by its distinct physicochemical properties, pharmacological effects, and diverse applications. It is a creamy white, water-soluble powder, devoid of common fenugreek phytochemicals such as saponins, flavonoids, alkaloids, and the amino acid 4-hydroxyisoleucine. FenuMat exhibits unique physico-mechanical characteristics and a highwater holding capacity, making it suitable for forming stable soft hydrogels with controlled release behaviour. Notably, these hydrogels revert to their powdered form upon dehydration, demonstrating their reversible nature. Additionally, FenuMat also possesses mucoadhesive properties, biocompatibility, and prebiotic potential, making it well suited for use as a natural self-emulsifying reversible hybrid-hydrogel system (N’SERH) for oral delivery of drugs and nutrients. FenuMat-mucilage has been effectively utilized for the preparation of taste-masked and sustained-release intestinal delivery systems for pungent chili extracts rich in capsaicinoids, with enhanced bioavailability and clinical efficacy [28]. It has also demonstrated efficiency in encapsulating gummy latexes, such as *Ferula asafoetida* and *Boswellia serrata* oleo-gum resins, into water-soluble microparticles, with better clinical efficacy [42], [43]. Additionally, it has been shown to facilitate the co-delivery of multiple phytonutrients, enhancing their bioavailability and therapeutic effectiveness [44].

Acute toxicity studies provide initial insights into the toxicological profile of a bioactive substance and aid in its classification and labeling. According to the Globally Harmonized System (GHS) of toxicity classification, substances with oral LD_50_ values in the range of 2000–5000 mg/kg body weight are considered safe [45]. Our findings demonstrated that the administration of FGS-002P at the highest tested dose of 2000 mg/kg body weight was well-tolerated in animals, as indicated by the absence of mortality, behavioural changes, alterations in food and water intake, body weight changes, and biochemical or hematological abnormalities. Necropsy, organ weight assessments, and histopathological evaluations further confirmed its safety. Based on these observations, FGS-002P can be classified under GHS Category 5, indicating a low toxicity profile with an LD_50_ > 2000 mg/kg body weight.

The 90-day repeated-dose subchronic toxicity study showed that the administration of FGS-002P at doses of 250, 500, and 1000 mg/kg body weight was well tolerated, with no side effects or adverse events. Necropsy findings, organ weights, and histopathological evaluations further supported the safety of FGS-002P at all the tested doses. Necropsy results revealed normal appearance of all examined organs and tissues, with no observable gross pathological changes in animals treated with 1000 mg/kg body weight. Additionally, histopathological analysis of various organs, including the liver, kidney, spleen, brain, lungs, and heart, demonstrated normal cellular architecture comparable to that of untreated control groups. Our observations clearly indicates that FGS-002P administration does not affect the normal physiology of internal organs. There were no changes in behaviour or clinical parameters, apart from the non-toxic variations in body weight, and food and water consumption.

In the high-dose recovery group (1000 mg/kg body weight), food and water intake remained normal, suggesting unaffected metabolic processes. The normal food and water intake, along with the absence of inflammation, mucosal damage, or haemorrhage in the gastrointestinal tract indicated a healthy digestive system in these animals. Our results were also in agreement with the previous toxicity studies on galactomannans isolated from guar gum [46]. Additionally, the body weight gain observed during the 90-days treatment period was found to be reversible in the recovery group, implying that the body weight changes induced by FGS-002P were not due to permanent organ damage or metabolic disorders.

Hematological and biochemical parameters provide further insights regarding the safety of a bioactive substance. The lack of significant variations in platelet, Hb, RBC, and WBC counts even at the highest dose of 1000 mg/kg body weight for 90 days showed the absence of hematological toxicity of FGS-002P, compared to the control group. Our results suggests that FGS-002P does not exert toxic effects on the hematopoietic system or induce systemic inflammation or immune suppression. Among the biochemical markers of safety, liver function markers are considered important due to their sensitivity. The liver function tests primarily assess hepatic function by monitoring bilirubin, albumin, total protein and the hepatic toxicity markers SGOT, SGPT and ALP. SGOT and SGPT are enzymes released into the blood stream when hepatocytes are damaged, indicating acute liver injury [47]. Elevated ALP levels are associated with cholestasis and bile duct obstruction. Other markers such as bilirubin, albumin and total protein reflect the liver’s excretory and synthetic functions. Additionally, maintenance of lipid profile (total cholesterol, LDL, HDL and triglyceride levels), is directly linked to liver health, since the liver plays a central role in lipid metabolism [48]. It has already been shown that fenugreek dietary fiber can inhibit the absorption of bile salts and cholesterol and this may be the underlying mechanism behind its hypolipidemic effect [14], [15], [16]. The fact that the supplementation with FGS-002P even at the highest dose of 1000 mg/kg body weight did not produce any significant variations in these markers, suggests that it does not impair liver function or lipid metabolism, thereby supporting its safety profile.

FGS-002P administration does not result in any significant change in serum urea, creatinine and uric acid levels in the current study. Creatinine, a marker widely used to assess renal function, is primarily eliminated through glomerular filtration by the kidneys and hence the damage in renal function, especially in glomerular filtration, will cause its elevation. Similarly, urea and uric acid, both nitrogenous waste products eliminated by the kidneys, accumulate in the blood when renal excretory function is compromised [49]. Therefore, the unchanged levels of these markers suggest that FGS-002P does not adversely affect the filtration, excretion, or overall functional capability of the kidneys.

Absence of mutagenicity is a critical component in the regulatory evaluation of novel dietary fibers, as it ensures the compound does not pose a risk of genetic damage or carcinogenicity with long-term consumption. Such evidence is necessary for confirming the safety profile of dietary ingredients intended for human use. The Bacterial reverse mutation test, commonly referred to as the AMES test, is a widely accepted genotoxicity assay used to assess the mutagenic potential of a substance. In the present study, the ability of F-GM to induce reverse mutations at the histidine loci was evaluated in four *S. typhimurium* strains- TA 98, TA 100, TA 1535 and TA 102- following standard testing protocols. Mutagenic substances can cause genetic alterations and may act as potential carcinogens. Some act as direct mutagens and some others are dependent on metabolic activation via microsomal enzymes, such as those found in the S9 fraction. F-GM was tested across a concentration range of 0.128–5 mg/plate, both with and without the S9 metabolic activation system. The results showed no signs of mutagenicity in any of the tested strains, under both conditions, indicating the absence of genotoxic potential. These findings are in line with a previous study involving low molecular weight fenugreek galactomannan [32].

Soluble dietary fiber in fenugreek has been reported to play a major role in delaying gastric emptying and glucose absorption, thereby contributing to its hypoglycemic activity [9], [10]. In our study, rats treated with 500 and 1000 mg/kg body weight of FGS-002P exhibited a decreasing trend in blood glucose levels, consistent with previous studies [50]. The observed hypoglycemic activity may be attributed to multiple mechanisms, including suppression of intestinal glucose absorption, inhibition of hepatic glucose production, enhancement of insulin sensitivity, and decline of oxidative stress, inflammation, and lipid peroxidation. Furthermore, the high mucoadhesive property of F-GM may prolong its retention at the site of action, allowing sustained interaction with intestinal enzymes and epithelial cells. This extended contact likely contributes to its hypoglycemic effects by further slowing glucose absorption. Moreover, the dense galactoside side chains in F-GM are associated with its pronounced cholesterol- lowering effects [8], [9], [10], highlighting the role of its molecular structure in mediating therapeutic benefits.

Our findings are consistent with previous toxicity studies on galactomannan derived from different sources such as tara gum, gur gum, and fenugreek [23], [46], [50]. No significant variations in biochemical parameters were observed even after the repeated administration of FGS-002P for 90 days, including at doses of 1000 mg/kg body weight tested in the present study. Earlier studies on acute, subchronic, and genotoxicity assessments of low molecular weight fenugreek galactomannan have reported an LD_50_ greater than 2000 mg/kg body weight [23], while a repeated-dose study for 90 days established a no-observed-adverse-effect level (NOAEL) at 1000 mg/kg body weight. These findings are in line with the observations of the present study. In addition to this, a prenatal developmental toxicity study in Wistar rats highlighted its protective effects against developmental toxicity [32]. Based on the present *in vivo* data, the galactomannan-rich fenugreek seed extract, FenuMat®, appears to be safe, with an estimated LD_50_ as > 2000 mg/kg body weight and a NOEAL of 1000 mg/kg body weight supporting the possibility of its potential use in human applications. Based on the NOEAL, the human equivalent dose is calculated and found to be 162 mg/kg body weight. [51].

The absence of reproductive, developmental, chronic toxicity, and carcinogenic data are the limitations of our present study. This work was designed as a preliminary and sub-chronic safety evaluation in Wistar rats in accordance with OECD 408 and 423 guidelines, mainly to evaluate the safety profile of FenuMat®. Consequently, parameters related to fertility, tetragenicity, and prenatal or postnatal effects, as well as long-term toxicity and carcinogenic potential, were not included within the current experimental scope. These aspects are, however, acknowledged as necessary for a comprehensive toxicological assessment. Future studies are planned to address these endpoints through extended and reproductive toxicity investigations to establish a more complete safety profile of FenuMat®.

## 5 Conclusion

This study evaluated the safety of FenuMat®, a proprietary fenugreek mucilage dietary fiber, through both acute (14 days) and subchronic (90 days) oral toxicity evaluations at doses of 250, 500, and 1000 mg/kg body weight. In the acute toxicity study, a high dose of 2000 mg/kg b.wt elicited no signs of toxicity. Similarly, the 90 days repeated-toxicity study showed no significant alterations in hematological, biochemical, or histological parameters, supporting its overall safety. Notably, a reduction in serum glucose levels at 500 and 1000 mg/kg body weight, along with a decline in LDL-cholesterol at 1000 mg/kg body weight, suggests potential hypoglycemic and hypolipidemic effects of the fiber. Histopathological analysis confirmed the absence of morphological abnormalities in vital organs. In conclusion, FenuMat®-fiber appears to be sae at doses up to 1000 mg/kg body weight per day, indicating its suitability for human use.

## CRediT authorship contribution statement

**S Syam Das:** Writing – review & editing, Writing – original draft. **Rani K Cherian:** Supervision, Software, Project administration, Investigation, Formal analysis. **Megha Kelenchery Ganesh:** Visualization, Supervision, Software, Project administration, Investigation, Formal analysis. **IM Krishnakumar:** Writing – review & editing, Methodology, Conceptualization. **Aryan Manu Kanjoormana:** Writing – review & editing.

## Ethics & regulatory declaration

All animal experiments were carried out in strict accordance with the ethical norms approved by the Institutional Animal Ethics Committee (IAEC) recognized by the Committee for the Purpose of Control and Supervision of Experiments on Animals (CPCSEA), Government of India (Registration No: CKL/TOX/IAEC/2023–2/202).

## Funding

Funding received from Akay Natural Ingredients Pvt Ltd, Cochin, Kerala, India.

## Declaration of Competing Interest

The authors declare the following financial interests/personal relationships which may be considered as potential competing interests: The authors declare the following conflict of interests. The test substance used in this study is a patented and registered by Akay Natural Ingredients Pvt Ltd, Cochin, Kerala, India as "FenuMat®". Syam Das Sivadasan (SDS) and Krishnakumar I M (KIM) belongs to Akay Natural Ingredients Pvt Ltd, Cochin, Kerala, India, who prepared the test substance, approved the protocol and provided financial support". Others have no conflict of interests

## Data Availability

Data will be made available on request.
