# Supplementary material for: Safety assessment of a proprietary fenugreek mucilage composition (FenuMat®): Acute and subchronic toxicity studies
Source: Toxicol Rep. 2025 Dec 18;16:102187. doi: 10.1016/j.toxrep.2025.102187 (PMC13711065; doi:10.1016/j.toxrep.2025.102187)
Supplement: Supplementary file 1 [file mmc1.docx]

**List of Figures**

Figure 1: Chemical structure of Fenugreek galactomannans

Figure 2A: Body weight of male rats during the administration of FGS-002P for 90-days and 28-days of the recovery period

Figure 2B: Body weight of female rats during the administration of FGS-002P for 90-days and 28-days of the recovery period

Figure 3A: Food intake of male during the administration of FGS-002P for 90-days and 28-days of the recovery period

Figure 3B: Food intake of female during the administration of FGS-002P for 90-days and 28-days of the recovery period

Figure 4A: Water intake of male rats during the administration of FGS-002P for 90-days and 28-days of the recovery period

Figure 4B: Water intake of female rats during the administration of FGS-002P for 90-days and 28-days of the recovery period

Figure 5A: Histopathological analysis of administration of FGS-002P for 90 days and 28 days of the recovery period in male Wistar rats by H&E staining. I: Control; II: Control R; III: 250 mg; IV: 500 mg; IV: 1000 mg: VI: HD R

Figure 5B: Histopathological analysis of administration of FGS-002P for 90 days and 28 days of the recovery period in female Wistar rats by H&E staining. I: Control; II: Control R; III: 250 mg; IV: 500 mg; IV:1000 mg: VI: HD R

Figure 1

Figure 2A

Figure 2B

Figure 3A

Figure 3B

Figure 4A

Figure 4B

Figure 5A

Figure 5B
