# Supplementary material for: Safety assessment of a proprietary fenugreek mucilage composition (FenuMat®): Acute and subchronic toxicity studies
Source: Toxicol Rep. 2025 Dec 18;16:102187. doi: 10.1016/j.toxrep.2025.102187 (PMC13711065; doi:10.1016/j.toxrep.2025.102187)
Supplement: Supplementary file 2 [file mmc2.docx]

**Supplementary Tables**

Table S1: Summary of Body Weight (g) and Body Weight Gain (%) [Acute toxicity study- 14 days]

| **Dose (mg/kg)** | **No. of Rats** | **Sex** | **Average Body weight on days** | | | **Body weight gain (%)** | |
| --- | --- | --- | --- | --- | --- | --- | --- |
|  |  |  | **Day 1** | **Day 7** | **Day 14** | **1 to 7** | **1 to 14** |
| 2000 | 1 | F | 215.00± 0.00 | 225.00± 0.00 | 235.00 ± 0.00 | 4.65 ± 0.00 | 9.30 ± 0.00 |
| 2000 | 4 | F | 220.00± 4.56 | 226.25 ± 5.54 | 233.75 ± 5.54 | 2.83 ± 1.10 | 6.26 ± 1.48 |

F= Female, Values are expressed as mean ± SEM

Table S2: Summary of food intake (g) [Acute toxicity study- 14 days]

| **Dose (mg/kg)** | **No. of Rats** | **Sex** | **Average Food intake (g)** | | |
| --- | --- | --- | --- | --- | --- |
|  |  |  | **1 to 7 days** | **8 to 14 days** | **1 to 14 days** |
| 2000 | 1 | F | 16.43 ± 0.92 | 12.86 ± 1.49 | 14.64 ± 0.98 |
| 2000 | 4 | F | 13.39 ± 0.73 | 14.29 ± 0.56 | 13.84 ± 0.46 |

F= Female, Values are expressed as mean ± SE

Table S3: Summary of Water Intake (ml) [Acute toxicity study- 14 days]

| **Dose (mg/kg)** | **No. of Rats** | **Sex** | **Average water intake (ml)** | | |
| --- | --- | --- | --- | --- | --- |
|  |  |  | **1 to 7 days** | **8 to 14 days** | **1 to 14 days** |
| 2000 | 1 | F | 17.57 ± 1.41 | 13.86 ± 1.08 | 15.71 ± 1.00 |
| 2000 | 4 | F | 15.71 ± 0.78 | 20.18 ± 0.64 | 17.95 ± 0.58 |

F= Female, Values are expressed as mean ± SEM

Table S4: Summary of Relative Organ Weight (g/100g bwt.) [Acute toxicity study- 14 days]

| **Dose (mg/kg)** | **No. of Rats** | **Sex** | **Liver** | **Kidney** | **Spleen** | **Heart** | **Lungs** | **Brain** | **Ovary** |
| --- | --- | --- | --- | --- | --- | --- | --- | --- | --- |
| 2000 | 1 | F | 3.38 ± 0.00 | 0.80 ± 0.00 | 0.17 ± 0.00 | 0.35 ± 0.00 | 0.88 ± 0.00 | 0.74 ± 0.00 | 0.09 ± 0.00 |
| 2000 | 2 | F | 3.87 ± 0.09 | 0.79 ± 0.03 | 0.18 ± 0.01 | 0.37± 0.02 | 0.75 ± 0.07 | 0.71 ± 0.01 | 0.13 ± 0.02 |

F= Female; Values are expressed as mean ± SEM
